# Platelet-Released Growth Factors Induce Differentiation of Primary Keratinocytes

**DOI:** 10.1155/2017/5671615

**Published:** 2017-07-20

**Authors:** Andreas Bayer, Mersedeh Tohidnezhad, Justus Lammel, Sebastian Lippross, Peter Behrendt, Tim Klüter, Thomas Pufe, Holger Jahr, Jochen Cremer, Franziska Rademacher, Regine Gläser, Jürgen Harder

**Affiliations:** ^1^Department of Heart and Vascular Surgery, University Hospital of Schleswig-Holstein, Campus Kiel, Arnold-Heller Straße 3, Haus 18, 24105 Kiel, Germany; ^2^Institute of Anatomy and Cell Biology, RWTH University of Aachen, Wendlingweg 2, 52072 Aachen, Germany; ^3^Department of Dermatology, University Hospital of Schleswig-Holstein, Campus Kiel, Rosalind-Franklin-Straße 7, 24105 Kiel, Germany; ^4^Department of Traumatology, University Hospital of Schleswig-Holstein, Campus Kiel, Arnold-Heller Straße 3, Haus 18, 24105 Kiel, Germany; ^5^Department of Orthopedics, Aachen University Hospital, Pauwelsstr. 30, 52074 Aachen, Germany

## Abstract

Autologous thrombocyte concentrate lysates, for example, platelet-released growth factors, (PRGFs) or their clinically related formulations (e.g., Vivostat PRF®) came recently into the physicians' focus as they revealed promising effects in regenerative and reparative medicine such as the support of healing of chronic wounds. To elucidate the underlying mechanisms, we analyzed the influence of PRGF and Vivostat PRF on human keratinocyte differentiation in vitro and on epidermal differentiation status of skin wounds in vivo. Therefore, we investigated the expression of early (keratin 1 and keratin 10) and late (transglutaminase-1 and involucrin) differentiation markers. PRGF treatment of primary human keratinocytes decreased keratin 1 and keratin 10 gene expression but induced involucrin and transglutaminase-1 gene expression in an epidermal growth factor receptor- (EGFR-) dependent manner. In concordance with these results, microscopic analyses revealed that PRGF-treated human keratinocytes displayed morphological features typical of keratinocytes undergoing terminal differentiation. In vivo treatment of artificial human wounds with Vivostat PRF revealed a significant induction of involucrin and transglutaminase-1 gene expression. Together, our results indicate that PRGF and Vivostat PRF induce terminal differentiation of primary human keratinocytes. This potential mechanism may contribute to the observed beneficial effects in the treatment of hard-to-heal wounds with autologous thrombocyte concentrate lysates in vivo.

## 1. Introduction

The optimal therapy of patients' chronic, hard-to heal wounds is difficult and often not successful. Many patients worldwide experience minor or even major extremity of amputation due to chronic wound complications. In general, treatment options include surgical procedures as well as the application of diverse wound dressings. In the past decade, autologous thrombocyte concentrates lysates, for example, platelet-released growth factors (PRGFs), or their clinically related formulations (e.g., Vivostat PRF) came into the focus of regenerative and reparative medicine because they contain a multitude of chemokines, cytokines, and growth factors and are therefore supposed to support healing of chronic or infected wounds [1–5]. It has been shown that PRGF has the opportunity to stimulate cell proliferation and tissue regeneration, to modify cell and tissue differentiation, and to support angiogenesis [6–13]. Although beneficial clinical effects of a local application of autologous thrombocyte concentrate lysates on the healing of chronic or complicated wounds in vivo have been described [14–16], little is known about possible mechanisms involved. Recently, we have shown that platelet-released growth factors induce the antimicrobial peptides human beta-defensin-2 in primary human keratinocytes indicating an enhancement of the epithelial barrier function by PRGF and Vivostat PRF treatment [17]. So far, further investigations on possible mechanisms involved are rare. Therefore, we examined a possible influence of PRGF on human keratinocyte differentiation by analyzing the influence of PRGF on the expression of keratin 1, keratin 10, transglutaminase-1, and involucrin in primary human keratinocytes as indicators of their differentiation status. Keratin 1 and keratin 10 are primarily expressed on keratinocytes of the stratum basale and spinosoum and are therefore used as markers for their early terminal differentiation [18, 19]. Involucrin and transglutaminase-1 are mainly expressed on mature human keratinocytes of the stratum granulosum and corneum and are regarded as indicators of the late terminal differentiation of human keratinocytes [20–23].

## 2. Materials and Methods

### 2.1. Preparation of PRGF

In general, PRGF used for one in vitro experiment was obtained from a single donor. PRGF was prepared from supernatants of freshly donated human thrombocyte concentrates (Institute of Transfusion Medicine, University of Schleswig-Holstein, Campus Kiel) derived from leucocyte-depleted haemapheresis according to the officially recommended practice (Richtlinien zur Gewinnung von Blut und Blutbestandteilen und zur Anwendung von Blutprodukten (Hämotherapie), Transfusionsgesetz, Bundesärztekammer, 2010). Thrombocyte concentration exceeded 2–4 × 10^11^ per concentrate (200–450 ml). It includes less than 1 × 10^6^ leucocytes and 3 × 10^9^ erythrocytes. To prepare the PRGF, the freshly donated thrombocyte concentrates were centrifuged for 10 minutes at 2000*g*. Afterwards, the thrombocyte pellet was washed twice with a sodium citrate buffer (0.11 mM, ph 5.5, 37°C) and centrifuged again for 10 min at 2000*g*. The thrombocytes were resuspended in half the volume of the initial thrombocyte concentrate volume using Keratinocyte Growth Medium 2 (PromoCell, Heidelberg, Germany) without supplements. The resuspended thrombocytes were stored on ice, lysed by ultrasound, and stored at −80°C for 24 hours. The next day, the suspension was thawn again, the ultrasound procedure was repeated, and the suspension stored again at −80°C for 24 hours. After repeated thawing, the suspension was centrifuged for 1 minute at 18.000*g*. The PRGF is the supernatant which was stored in aliquots at −20°C.

### 2.2. Preparation and Application of Vivostat PRF (Platelet-Rich Fibrin)

To prepare Vivostat PRF, 120 mL fresh blood from one patient was transferred to the so-called preparation unit and subsequently processed by the processor unit to obtain 5-6 mL of the Vivostat PRF product containing approximately 7 times the base level of the donor's blood thrombocyte concentration (>1 million/*μ*L) and fibrin (average concentration of 18.1 mg/mL) (for details, see http://www.vivostat.com). The Vivostat PRF was sprayed on the wound surface to completely cover the wound area using the Vivostat spraypen® (see also http://www.vivostat.com).

### 2.3. Culture and Stimulation of Primary Human Keratinocytes

We cultured foreskin-derived primary human keratinocytes pooled from different individuals (PromoCell) in Keratinocyte Growth Medium 2 (KGM-2, PromoCell, Heidelberg, Germany) at 37°C in a humidified atmosphere with 5% CO_2_. The keratinocytes were seeded for stimulatory experiments in 12-well tissue culture plates (BD Biosciences, Franklin Lakes, New Jersey). When they reached a 90–100% confluence, we thawed the frozen PRGF, diluted it to the indicated concentrations (PRGF 1 : 50, PRGF 1 : 20, and PRGF 1 : 10) with KGM-2, and stimulated primary keratinocytes.

For EGFR- or IL-6 receptor-blocking experiments, we used the EGFR-blocking antibody cetuximab (Merck, Darmstadt, Germany) or the IL-6 receptor-blocking antibody tocilizumab (Hoffmann-La Roche, Basel, Switzerland) at a concentration of 20 *μ*g/mL and 50 *μ*g/mL, respectively. After stimulation, the cells were washed with 1 mL per well of PBS followed by the isolation of the RNA.

### 2.4. RNA-Isolation and cDNA Synthesis

We harvested keratinocytes from one well of a 12-well plate and lysed them using 500 *μ*L Crystal RNAmagic reagent. Total RNA was isolated according to the supplier's protocol (BioLab Products, Bebensee, Germany). The concentration of isolated total RNA was photometrically determined using a NanoDrop device (Peqlab, Erlangen, Germany). 1 *μ*g of total RNA was reversely transcribed to cDNA using oligo-dT-primers and 50 Units Maxima Reverse Transcriptase (Thermo Fisher Scientific, Waltham, USA) according to the manufacturer's protocol.

### 2.5. Real-Time PCR

We performed real-time PCR analyses in a fluorescence-temperature cycler (StepOnePlus, Life Technologies) as previously described [24]. The following intron spanning primers were used: keratin 1: 5′-CTT CTT CAG CCC CTC AAT GT-3′ (forward primer) and 5′-GTA CCT GGT TCT GCT GCT CC-3′ (reverse primer); keratin 10: 5′-TGA AAA GCA TGG CAACTC AC-3′ (forward primer) and 5′-TGT CGA TCT GAA GCA GGA TG-3′ (reverse primer); involucrin: 5′-CTG CCT CAG CCT TAC TGT GA-3′ (forward primer) and 5′-GGA GGA GGA ACA GTC TTG AGG-3′ (reverse primer); and transglutaminase-1: 5′-CCC TCA CCA ATG TCG TCT TC-3′ and 5′-TCA CTG TTT CAT TGC CTC CA-3′. We obtained standard curves by serial dilutions of cDNA and normalized all quantifications to the housekeeping gene RPL38 (ribosomal protein L38) using the primer pair: 5′-TCA AGG ACT TCC TGC TCA CA-3′ (forward primer) and 5′-AAA GGT ATC TGC TGC ATC GAA-3′ (reverse primer). Relative expression is given as a ratio between expression of the specific gene and expression of the housekeeping gene RPL38.

### 2.6. Analyses of the Influence of Vivostat PRF on the Epidermal Expression of Keratin 1, Keratin 10, Transglutaminase-1, and Involucrin In Vivo

This study was conducted with human in vivo samples that have been already used in a previous study [17]. Briefly, we set bilateral gluteal wounds in five male test persons by punch biopsy (Ø 4 mm) after local anesthesia and treated the freshly generated wounds with either NaCl 0.9% (left) or Vivostat PRF (right) followed by the application of occlusive wound dressings (Biatain®, Coloplast, Germany) on the treated wounds. After 5 days, we removed the dressing and the dried wound exudate by a sterile moistened compress and repeated the treatment as mentioned above. After 10 days, dressings were removed and bilateral wound areas were resected by punch biopsies (Ø 6 mm). RNA was isolated using RNAeasyKit (Qiagen, Hilden, Germany), and reverse transcription of the RNA and real-time PCR was performed as described above. This pilot study was approved by the University Committee for Ethical Affairs Kiel (AZ A 115/13) in accordance with the Helsinki guidelines. All participants included in this investigation provided written informed consent.

### 2.7. Statistics

GraphPad Prism 6.07 was used for statistical analysis and was carried out by Student's *t*-test or one-way ANOVA with Tukey's multiple comparison test. A *P* value of <0.05 was considered statistically significant.

## 3. Results

### 3.1. Dose-Dependent Influence of PRGF on the Expression of Differentiation Markers in Primary Keratinocytes

**Figure 1:**
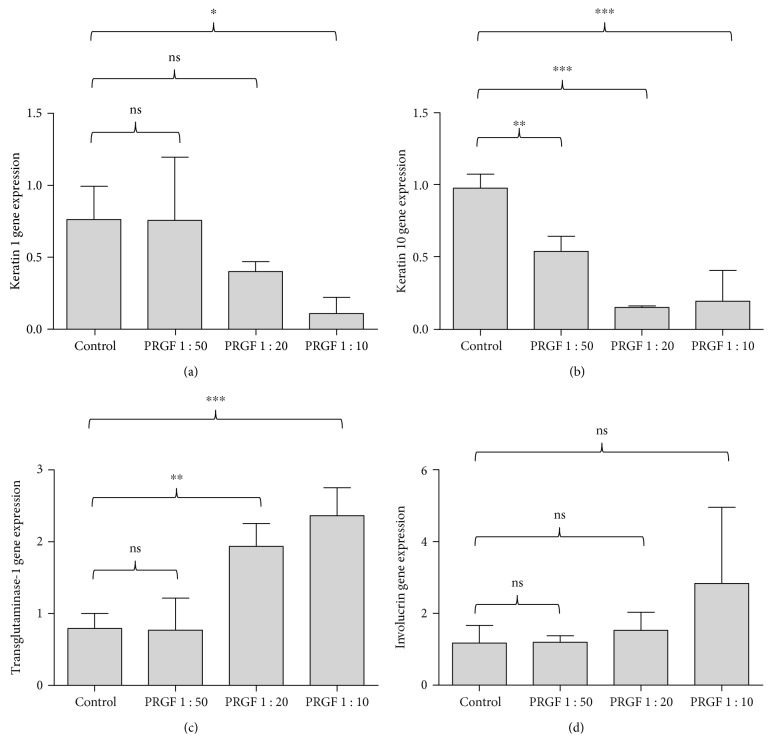
PRGF dose dependently influences the expression of differentiation markers in primary keratinocytes. PRGF stimulation of primary keratinocytes over 24 hours caused a dose-dependent decrease of keratin 1 (a) and keratin 10 (b) gene expression paralleled by a dose-dependent increase of transglutaminase-1 (c) and involucrin (d) gene expression (real-time PCR analysis, ^∗^*P* < 0.05, ^∗∗^*P* < 0.01, ^∗∗∗^*P* < 0.001, ns = not significant, one-way ANOVA with Tukey's multiple comparison test).

To investigate a possible influence on the keratin 1, keratin 10, transglutaminase-1, and involucrin gene expression, we stimulated the keratinocytes with different concentrations of PRGF for 24 hours. PRGF stimulation caused a significant decrease of keratin 1 (Figure 1(a)) and keratin 10 (Figure 1(b)) gene expression paralleled by a significant increase of transglutaminase-1 (Figure 1(c)) and an insignificant increase of involucrin gene expression (Figure 1(d)) in the stimulated keratinocytes.

### 3.2. PRGF Time Dependently Influences the Expression of Differentiation Markers in Primary Human Keratinocytes

**Figure 2:**
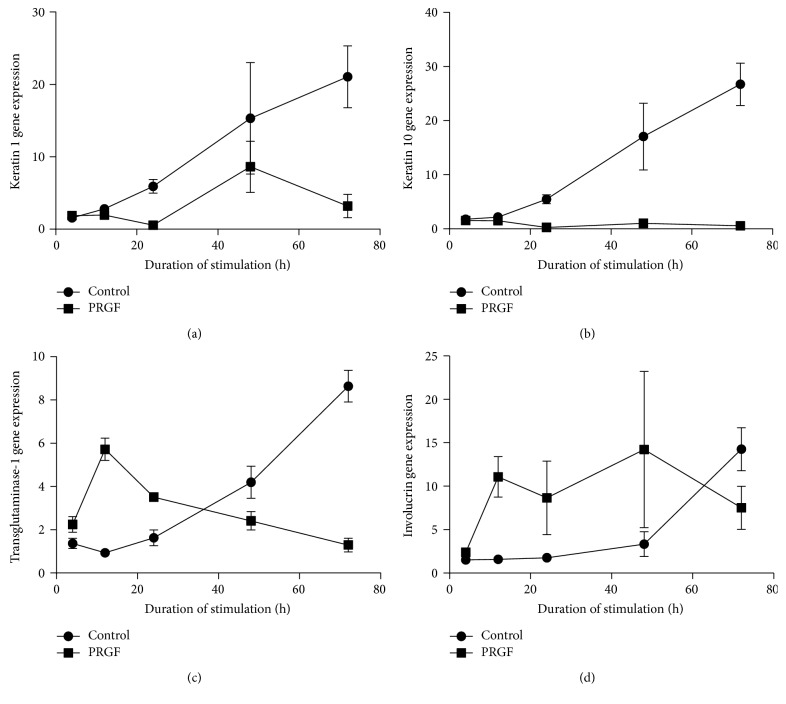
Time-dependent influence of PRGF on the expression of differentiation markers. PRGF stimulation (1 : 10) of primary keratinocytes caused a time-dependent decrease of keratin 1 (a) and keratin 10 (b) gene expression paralleled by a time-dependent increase of transglutaminase-1 (c) and involucrin (d) gene expression.

We aimed to analyze the time kinetic of the PRGF treatments' influence on the gene expression of keratin 1, keratin 10, transglutaminase-1, and involucrin in primary human keratinocytes. Therefore, we measured gene expression of these differentiation markers after 4, 12, 24, 48, and 72 hours of PRGF stimulation. Detection of keratin 1 and keratin 10 gene expression revealed a marked decrease after 24, 48, and 72 hours of PRGF stimulation (Figures 2(a) and 2(b)). In contrast, transglutaminase-1 (Figure 2(c)) and involucrin (Figure 2(d)) gene expression was markedly induced in PRGF-stimulated primary human keratinocytes after 12 and 24 hours of stimulation.

### 3.3. PRGFs from Different Donors Exhibit Similar Influences on the Expression of Differentiation Markers in Primary Human Keratinocytes

**Figure 3:**
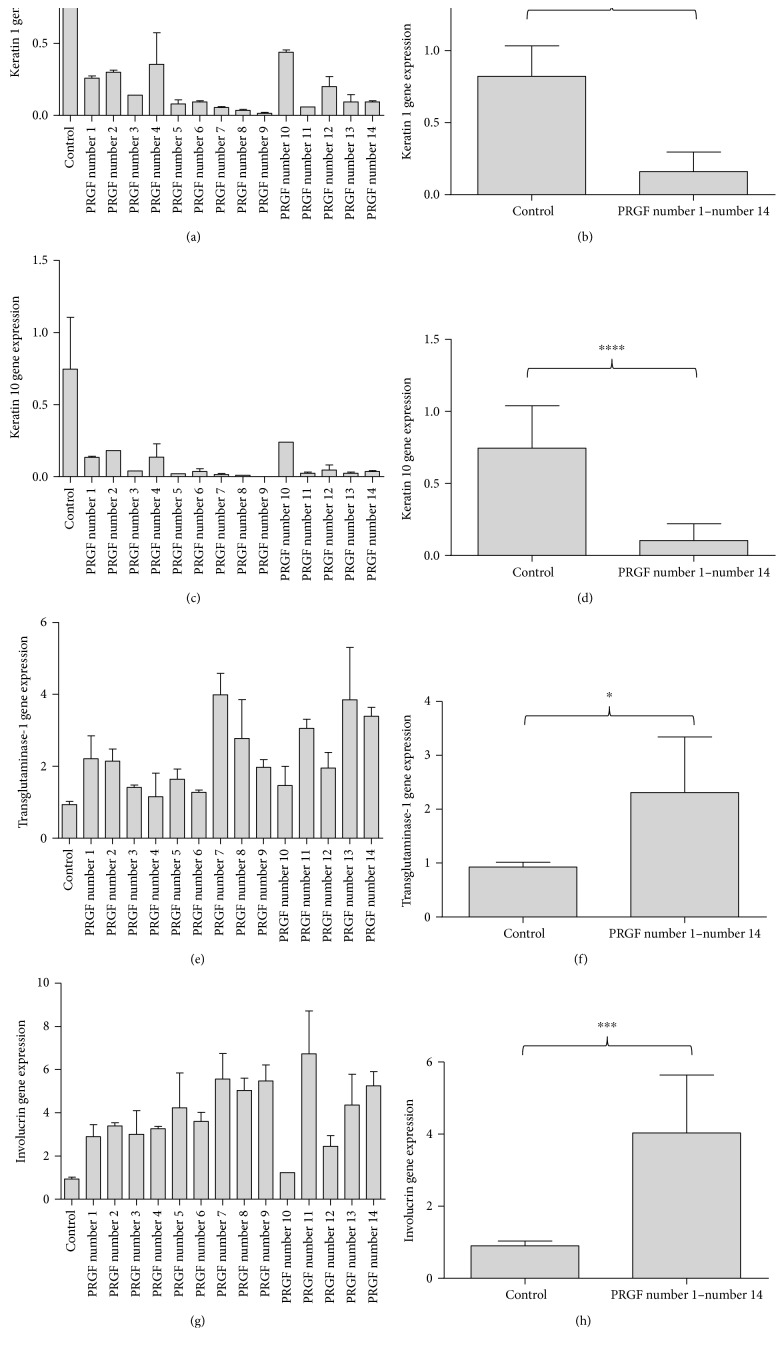
PRGF from different donors influenced gene expression of differentiation markers in a similar manner. Analyzing the capacity of PRGF from 14 different donors (PRGF number 1–PRGF number 14), we observed a strong and interindividually different effect of the used PRGF on the expression of keratin 1, keratin 10, transglutaminase-1, and involucrin in primary keratinocytes (a, c, e, g). The mean values of all 14 PRGFs analyzed (b, d, f, h) revealed a significant influence of the PRGF treatment on the gene expression of the analyzed differentiation markers in primary human keratinocytes (real-time PCR analysis, ^∗^*P* < 0.05, ^∗∗∗^*P* < 0.001, ^∗∗∗∗^*P* < 0.0001, Student's *t*-test).

In order to investigate the possible interindividual differences of PRGF on the expression of differentiation markers, we analyzed the influence of PRGF from fourteen different donors on the keratin 1, keratin 10, transglutaminase-1, and involucrin gene expression in primary human keratinocytes. These experiments revealed that all 14 PRGF preparations derived from different donors resulted in a reduced gene expression of keratin 1 (Figures 3(a) and 3(b)) and keratin 10 (Figures 3(c) and 3(d)) whereas gene expression of transglutaminase-1 (Figures 3(e) and 3(f)) and involucrin (Figures 3(g) and 3(h)) was induced.

### 3.4. The Epidermal Growth Factor Receptor (EGFR) Influences the Expression of Differentiation Markers in Primary Human Keratinocytes Treated with PRGF

**Figure 4:**
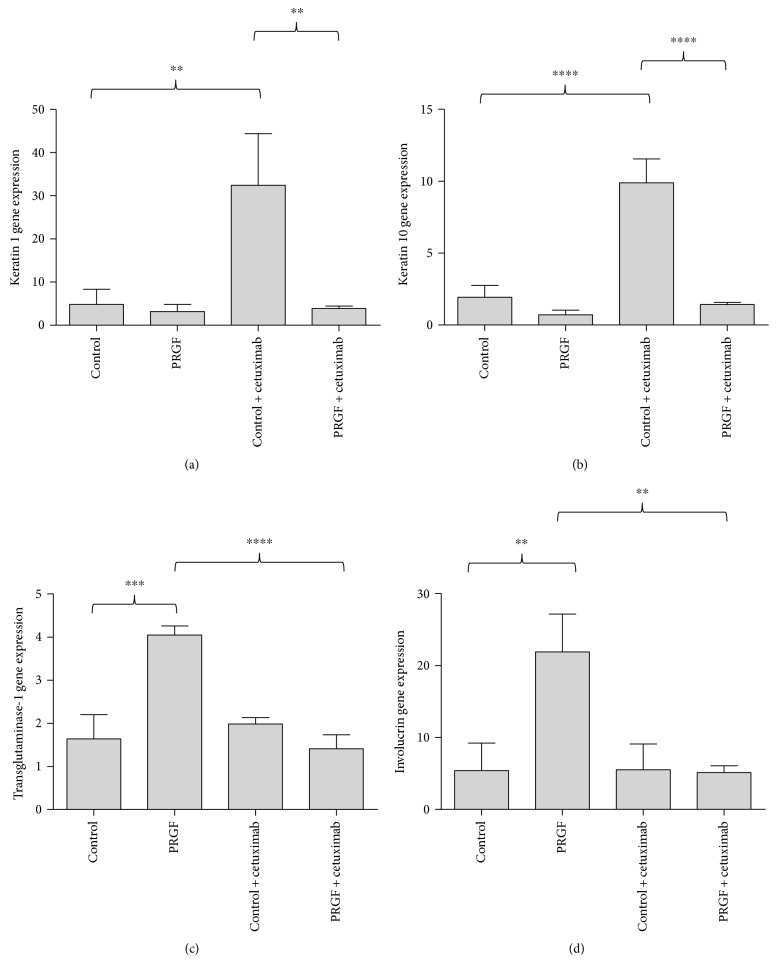
The epidermal growth factor receptor (EGFR) partially mediated the keratin 1, keratin 10, transglutaminase-1, and involucrin gene expression in keratinocytes stimulated with PRGF. PRGF stimulation of primary human keratinocytes caused a reduced keratin 1 and keratin 10 gene expression and an induced transglutaminase-1 and involucrin gene expression. Costimulation with the EGFR-blocking antibody cetuximab revealed that these effects were mediated via the EGFR (real-time PCR analysis, ^∗∗^*P* < 0.01, ^∗∗∗^*P* < 0.001, ^∗∗∗∗^*P* < 0.0001, one-way ANOVA with Tukey's multiple comparison test).

To examine the underlying signal transduction pathways of the PRGF-mediated keratin 1, keratin 10, transglutaminase-1, and involucrin gene expression in primary human keratinocytes, we analyzed the influence of the epidermal growth factor receptor (EGFR) using a specific monoclonal EGFR-blocking antibody (cetuximab). Treatment of the keratinocytes with cetuximab significantly induced the gene expression of keratin 1 (Figure 4(a)) and keratin 10 (Figure 4(b)), but this induction was inhibited by PRGF. In contrast, the inhibition of the EGFR using cetuximab did not induce transglutaminase-1 and involucrin expression but resulted in a significant decrease of PRGF-induced transglutaminase-1 (Figure 4(c)) and involucrin (Figure 4(d)) gene expression.

### 3.5. The Influence of PRGF on the Expression of Differentiation Markers Does Not Involve the Interleukin-6 Receptor (IL-6R)

**Figure 5:**
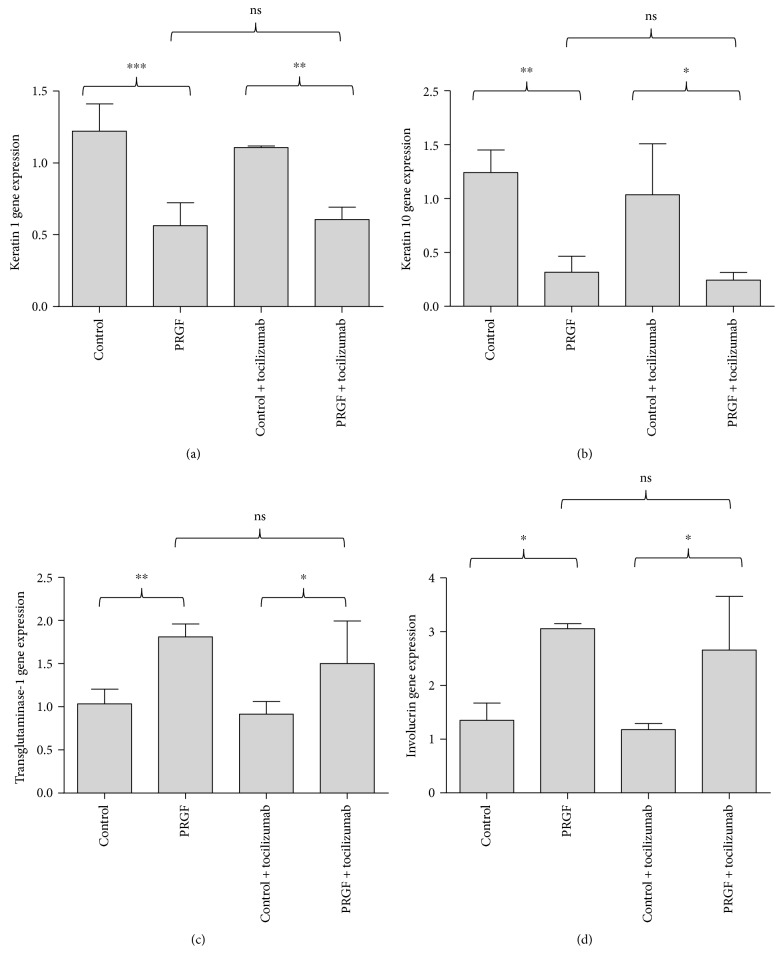
The IL-6 receptor is not involved in the PRGF-mediated modulation of differentiation markers in keratinocytes. PRGF stimulation of primary human keratinocytes caused a reduced keratin 1 and keratin 10 gene expression and an induced transglutaminase-1 and involucrin gene expression. Costimulation with the IL-6 receptor-blocking antibody tocilizumab revealed that these effects were not mediated via the IL-6 receptor (real-time PCR analysis, ^∗^*P* < 0.05, ^∗∗^*P* < 0.01, ^∗∗∗^*P* < 0.001, ns = not significant, one-way ANOVA with Tukey's multiple comparison test).

We have recently shown that PRGF treatment induced IL-6 in primary human keratinocytes already after 4 hours. In addition, the PRGF-mediated induction of the antimicrobial peptide hBD-2 partially depended on IL-6 signaling [17]. Therefore, we sought to determine whether IL-6 signaling plays a role in the expression of differentiation markers in keratinocytes treated with PRGF. To this end, we blocked the IL-6 receptor with the IL-6 receptor-neutralizing antibody tocilizumab and stimulated the keratinocytes with PRGF. These experiments revealed a significant decrease of keratin 1 (Figure 5(a)) and keratin 10 (Figure 5(b)) gene expression in PRGF-treated keratinocytes that seemed to be independent from the IL-6R. Similarly, the significant PRGF-mediated induction of transglutaminase-1 (Figure 5(c)) and involucrin (Figure 5(d)) was not influenced by blocking the IL-6R.

### 3.6. PRGF Induced Morphological Changes in Primary Human Keratinocytes In Vitro

**Figure 6:**
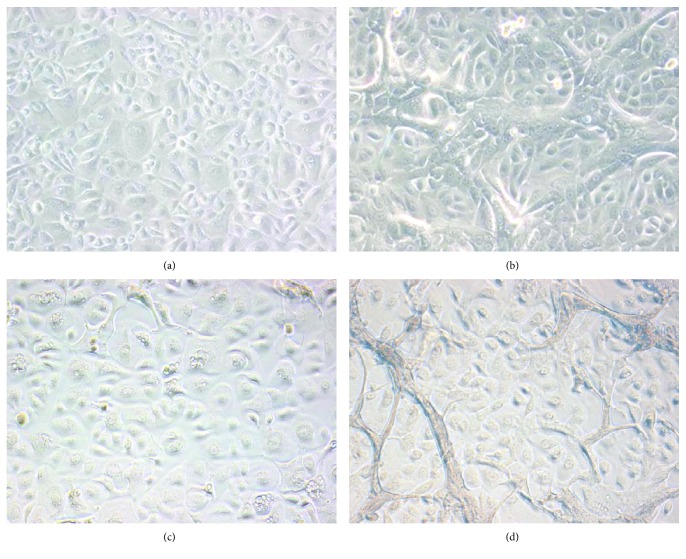
PRGF-treated primary human keratinocytes display morphologic features typical for keratinocytes in their late terminal differentiation phase. During all in vitro experiments, PRGF-treated primary human keratinocytes revealed a morphology that is a characteristic for keratinocytes undergoing terminal differentiation such as growth in several layers and loss of distinct cell borders. Shown are untreated primary human keratinocytes cultured for 24 hours (a) and 48 hours (c) compared to primary human keratinocytes treated with PRGF (1 : 10) for 24 hours (b) and 48 hours (d).

Microscopic observations during all in vitro experiments were striking. Whereas untreated primary human (control) keratinocytes (Figures 6(a) and 6(c)) displayed a cell and intercellular morphology typical for keratinocytes in their early differentiation status, PRGF-treated primary human keratinocytes presented the typical morphology of mature human keratinocytes in their late terminal differentiation status (Figures 6(b) and 6(d)).

### 3.7. Treatment of Human Cutaneous Wounds with Vivostat PRF Induced the Gene Expression of Involucrin and Transglutaminase-1 In Vivo

**Figure 7:**
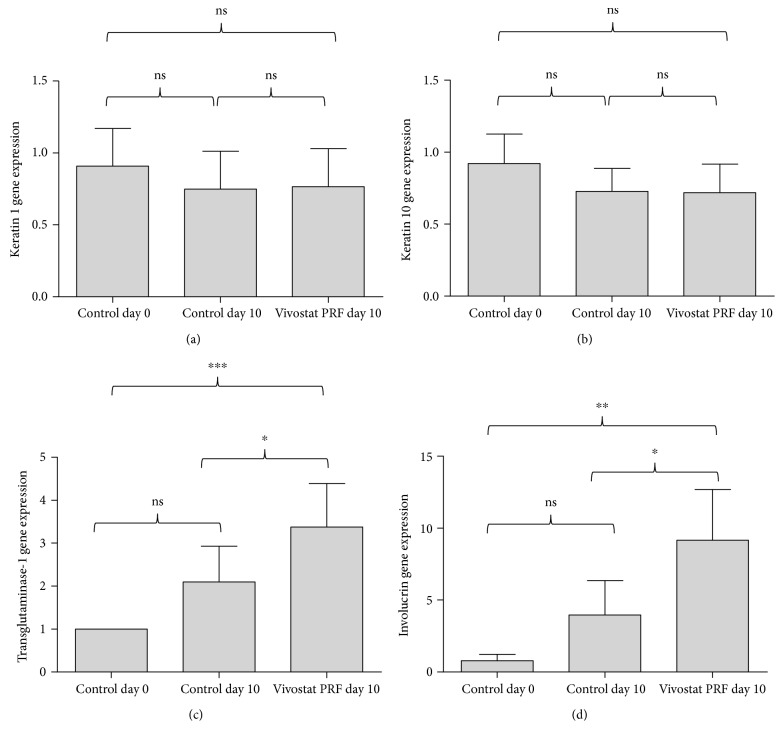
Vivostat PRF treatment caused a significant transglutaminase-1 and involucrin gene induction in vivo. In our in vivo study, we observed a significant induction of transglutaminase-1 and involucrin gene expression in Vivostat PRF-treated keratinocytes. Vivostat PRF treatment caused an insignificant decrease of keratin 1 and keratin 10 gene expression in human keratinocytes in vivo (real-time PCR analysis, ^∗^*P* < 0.05, ^∗∗^*P* < 0.01, ^∗∗∗^*P* < 0.001, ns = not significant, one-way ANOVA with Tukey's multiple comparison test).

To investigate if our in vitro data could be transferred into the in vivo setting, we performed the above mentioned human in vivo study. This study revealed a significant transglutaminase-1 (Figure 7(c)) and involucrin (Figure 7(d)) gene induction in the wounds treated with Vivostat PRF. Keratin 1 (Figure 7(a)) and keratin 10 (Figure 7(b)) gene expression was slightly but not significantly reduced by Vivostat PRF treatment.

## 4. Discussion

Chronic lower extremity skin ulcers affect about 3% of the population in Western countries and cause an immense personal, financial (approximately 2.5% of total healthcare budgets in Europe and America), and social burden [25]. Despite optimal causal and topical therapy, wound healing remains often difficult and unsuccessful. One new therapeutic option for the therapy of chronic wounds is the local application of an autologous thrombocyte concentrate lysate (e.g., Vivostat PRF). In general, thrombocytes play a major role during the initial uncomplicated wound healing process through their release of a multitude of cytokines, chemokines, and growth factors that initiate and coordinate the complex wound healing process [1–5]. For this reason, thrombocyte concentrate lysates are supposed to have the opportunity to optimize the unfavorable local wound milieu and support wound healing [26–28]. Due to their regenerative, reparative, and angiogenetic potential, thrombocyte concentrate lysates are recently used in many medical disciplines [9, 26, 29–33]. In the context of chronic lower extremity ulcers, Steenvoorde et al. described the use of autologous platelet-rich fibrin on a range of hard-to-heal wounds which caused complete wound healing or a significant reduction in wound diameter in the majority of treated patients [14]. Despite these positive clinical experiences with Vivostat PRF for the therapy of chronic or complicated wounds, little is known about possible mechanisms involved [12, 26–28].

Recently, we have demonstrated that thrombocyte concentrate lysates induce the antimicrobial peptide human beta-defensin-2 in keratinocytes [17] indicating an improved cutaneous innate defense function as one explanation for the observed positive clinical effects of autologous thrombocyte concentrate lysates on wound healing. We now aimed to investigate if thrombocyte concentrate lysates may also directly affect the formation of the epidermal barrier. In particular, we were interested to evaluate a potential influence on the epidermal differentiation process. Therefore, we analyzed gene expression patterns of early differentiation markers (keratin 1 and keratin 10) and late differentiation markers (transglutaminase-1 and involucrin) in keratinocytes [18–23, 34].

We observed that in vitro treatment of primary keratinocytes with PRGF pronounced late differentiation processes in primary human keratinocytes as documented by a PRGF-mediated decrease of keratin 1 and keratin 10 gene expression paralleled by an increase of transglutaminase-1 and involucrin gene expression. These effects were concentration dependent and time dependent with maximal effects seen after 12–24 hours of stimulation which is in concordance with data from Liew and Yamanishi [35] reporting that transglutaminase-1 gene expression peaked after 16 hours of 12-O-tetradecanoylphorbol-13-acetate- (TPA-) induced differentiation in keratinocytes. Thus, one may speculate that gene expression of transglutaminase-1 is induced during the differentiation process and decreased when the terminal differentiation phase is reached.

Accordingly, we observed in our in vivo study a significant gene induction of involucrin and transglutaminase-1 in Vivostat PRF-treated skin, thus translating the in vitro findings in the in vivo setting. In concordance with these cell culture and in vivo data, microscopic analyses revealed that PRGF-treated human keratinocytes—unlike untreated keratinocytes—displayed a morphology typical for late terminal differentiated keratinocytes. Taken together, these experiments identified PRGF and Vivostat PRF as a potent inducer of human keratinocyte differentiation.

To investigate possible mechanisms involved, we analyzed the underlying signal transduction pathways. As keratinocyte differentiation was demonstrated to be EGFR dependent [36–38], we analyzed the influence of the EGFR on the PRGF-mediated alteration of keratin 1, keratin 10, transglutaminase-1, and involucrin gene expression in human keratinocytes by blocking the EGFR via a monoclonal antibody (cetuximab). These studies demonstrated that the PRGF-mediated decrease of keratin 1 and keratin 10 gene expression as well as the increase of transglutaminase-1 and involucrin gene expression in keratinocytes—and thus the PRGF-induced keratinocytes differentiation—were predominantly EGFR mediated. In line with these results, it has been reported that the EGFR ligand HB-EGF, which is upregulated in the margin of skin wounds, induced involucrin gene expression in keratinocytes, whereas keratin 10 gene expression was downregulated [39]. These data suggest that EGFR ligands present in PRGF and Vivostat PRF activate the EGFR in keratinocytes leading to the observed involucrin and decreased keratin 10 expression. It has been reported that EGFR activation and signaling improve the integrity of the skin barrier by enhancing terminal keratinocyte differentiation and the cross-linking activity of transglutaminases [37]. Thus, PRGF may facilitate the reconstitution of an intact skin barrier by promoting terminal differentiation of the keratinocytes.

In former experiments, we detected a strong expression of IL-6 in primary human keratinocytes already after 4 hours of PRGF treatment [17]. In addition, IL-6 has been reported to negatively affect terminal keratinocytes differentiation [40]. Therefore, we asked if IL-6 may have an influence on the observed PRGF-mediated induction of keratinocyte differentiation. To address this question, we used tocilizumab, a monoclonal antibody directed against the IL-6-receptor (IL-6R), to inhibit the IL-6 signaling pathway. However, tocilizumab had no significant influence on the expression of differentiation markers in PRGF-treated keratinocytes indicating that IL-6 signaling plays no role in this process.

## 5. Conclusion

We demonstrated that PRGF stimulation of primary keratinocytes and Vivostat PRF application on artificially generated human skin wounds cause an accelerated differentiation process of primary human keratinocytes that may—in addition to the described induction of antimicrobial peptides [17]—contribute to the observed beneficial effects in the treatment of hard-to-heal wounds with autologous thrombocyte concentrate lysates in vivo.
